# Reproducing Patient-Specific Hemodynamics in the Blalock–Taussig Circulation Using a Flexible Multi-Domain Simulation Framework: Applications for Optimal Shunt Design

**DOI:** 10.3389/fped.2017.00078

**Published:** 2017-04-26

**Authors:** Christopher J. Arthurs, Pradyumn Agarwal, Anna V. John, Adam L. Dorfman, Ronald G. Grifka, C. Alberto Figueroa

**Affiliations:** ^1^Division of Imaging Sciences and Biomedical Engineering, King’s College London, King’s Health Partners, St. Thomas’ Hospital, London, UK; ^2^Department of Biomedical Engineering, University of Michigan, Ann Arbor, MI, USA; ^3^Department of Pediatric Cardiology, University of Michigan Health System, Ann Arbor, MI, USA; ^4^Department of Surgery, University of Michigan, Ann Arbor, MI, USA

**Keywords:** hypoplastic left heart syndrome, Blalock–Taussig shunt, computational hemodynamics, simulation, multi-domain, CRIMSON

## Abstract

For babies born with hypoplastic left heart syndrome, several open-heart surgeries are required. During Stage I, a Norwood procedure is performed to construct an appropriate circulation to both the systemic and the pulmonary arteries. The pulmonary arteries receive flow from the systemic circulation, often using a Blalock–Taussig (BT) shunt between the innominate artery and the right pulmonary artery. This procedure causes significantly disturbed flow in the pulmonary arteries. In this study, we use computational hemodynamic simulations to demonstrate its capacity for examining the properties of the flow through and near the BT shunt. Initially, we construct a computational model which produces blood flow and pressure measurements matching the clinical magnetic resonance imaging (MRI) and catheterization data. Achieving this required us to determine the level of BT shunt occlusion; because the occlusion is below the MRI resolution, this information is difficult to recover without the aid of computational simulations. We determined that the shunt had undergone an effective diameter reduction of 22% since the time of surgery. Using the resulting geometric model, we show that we can computationally reproduce the clinical data. We, then, replace the BT shunt with a hypothetical alternative shunt design with a flare at the distal end. Investigation of the impact of the shunt design reveals that the flare can increase pulmonary pressure by as much as 7% and flow by as much as 9% in the main pulmonary branches, which may be beneficial to the pulmonary circulation.

## 1 Introduction

Each year in the United States, 1 out of every 4,344 babies are born with hypoplastic left heart syndrome (HLHS) (1). Soon after birth, they develop respiratory distress, elevated heart rate, and hypoxemia. A multi-stage surgical program is required to construct pathways for blood flow to both the systemic and pulmonary circulations using only the single (right) ventricle. The Norwood procedure is the first stage of the surgical repair and is performed within the first week of life. The main pulmonary artery is detached from the branches, then connected to the hypoplastic aorta, creating a single arterial vessel. Then, a cylindrical 3.5- or 4.0-mm diameter tube, known as the Blalock–Taussig (BT) shunt, is placed between the innominate artery and the main pulmonary artery (MPA), allowing blood to supply the pulmonary circulation. This BT shunt operation is followed by two additional procedures, the Glenn procedure (performed at 4–6 months of age), and the Fontan procedure (performed at 18–36 months of age). During the Glenn procedure, the BT shunt is disconnected from the MPA and replaced with flow from the superior vena cava. Even in the best cases, the Norwood procedure can have several complications, including low cardiac output, arrhythmias, respiratory insufficiency, and stenosis of the pulmonary arteries or aorta (2). These complications can be affected by the function of the shunt. The first stage (Norwood) is crucial, as any complication can be magnified at subsequent operations, leading to increased morbidity and mortality. Currently, the mortality rate of the Norwood procedure is 15–25% (2). Over the years, the Norwood procedure has been refined. However, in spite of the refinements, there remain several shortcomings, such as the inability to adapt to the growing child, a high incidence of stenosis developing at the site of the pulmonary artery anastomosis, and even complete shunt occlusion. If stenosis develops at the pulmonary artery anastomosis, this markedly alters the amount and velocity of blood flow delivered to the pulmonary circulation, leading to adverse hemodynamics and propensity for developing progressive stenosis, all of which can add to morbidity and mortality. Therefore, there is interest in applying the tools of computational hemodynamics to this problem (3, 4). In this study, we investigate the computational reproduction of the hemodynamics, following BT shunt placement, and the impact of an alternative shunt design. To do this, we create a complex, patient-specific circulatory model, designed to reproduce the hemodynamic data recorded in a 4-month-old HLHS patient. We utilized our in-house geometric modeling, closed-loop circulatory design, and Navier–Stokes computational hemodynamics simulation package (5–7). These tools allow us to develop highly customizable lumped parameter model circuits that can capture complex facets of the patient’s presentation, including aortic atresia and mitral stenosis, as well as a large atrial septal defect and evidence of bilateral aortopulmonary collateralization with discrete collaterals to the right lung. Then, we use the model to consider an alternative, flared design for the BT shunt.

## 2 Materials and Methods

### 2.1 Patient Data

Clinical data were acquired for a 4-month-old child who was born with hypoplastic left heart syndrome. The patient had undergone a Stage 1 Norwood procedure at 5 days of age, in which the innominate artery was connected to the MPA via a 3.5 mm diameter modified BT shunt. At 4 months of age, during pre-hemi-Fontan cardiac catheterization, the pulmonary arterial pressures were measured using left and right pulmonary venous wedge pressure measurements. During the same procedure, pressures were measured in the ascending and descending aorta. In an additional pre-hemi-Fontan research procedure, cardiac MRI and MRA were performed, providing data on ventricular end-systolic and end-diastolic volumes and flows through the superior and inferior vena cava, the ascending and descending aorta, the left and right pulmonary arteries (LPA; RPA), and veins (LPV; RPV).

### 2.2 Geometric Modeling and Discretization

**Figure 1:**
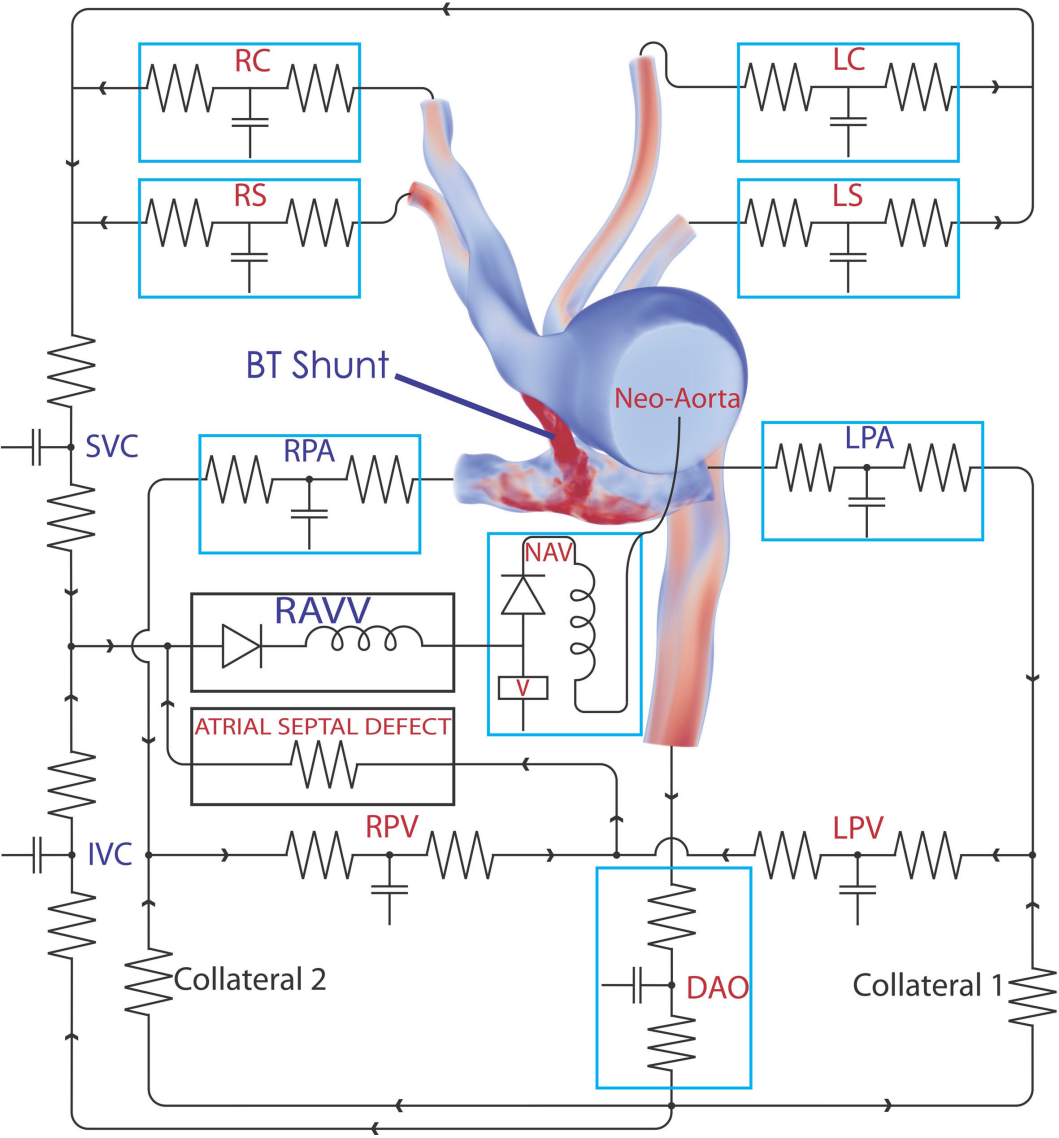
**The complete 3D–0D multi-domain model, including the closed-loop LPN circuit representing the physiological circulation of this patient**. The single functioning ventricle, atrial septal defect, and collateralization are all modeled. A representative volume rendering of the velocity field is shown at one instant in time; red indicates high velocity and blue low velocity. Abbreviations: RC, right common carotid; RS, right subclavian; LC, left common carotid; LS, left subclavian; SVC, superior vena cava; IVC, inferior vena cava; RPV, right pulmonary vein; LPV, left pulmonary vein; DAO, descending aorta; LPA, left pulmonary artery; RPA, right pulmonary artery; RAVV, right atrio-ventricular valve; V, ventricle; NAV, neo-aortic valve. See Tables 1–3 for all component parameter values.

A patient-specific computer-aided design (CAD) model of the arteries of the chest region was segmented from MRI image data of the patient using the geometric modeling tools within CRIMSON. This model contains the following vessels: ascending and descending aorta, left and right subclavian arteries, left and right common carotid arteries, left and right pulmonary artery, and surgically placed BT shunt. The model consists of one inlet (i.e., the ascending aorta) and seven outlets (i.e., all other vessels described above). The extent of the model is sufficient to capture complex blood flow in the BT shunt and the pulmonary artery anastomosis. The hemodynamic description of this circulation is displayed in Figure 1. In preparation for finite element simulation, a linear tetrahedral finite element mesh was generated, consisting of 231,484 nodes and 1,215,138 elements. A preliminary simulation was performed using this mesh, and an adaptive field-based mesh refinement strategy, striving to ensure the mesh was sufficiently refined for our studies was subsequently employed (8). This approach produced additional meshes consisting of 659,851 nodes and 3,722,358 elements, and finally 872,046 nodes and 4,993,559 elements; the final reported results use this finest mesh. All simulations were performed using a time-step of 0.1 ms, and the time integration scheme utilized, the generalized *α*-method, provided second-order accuracy and unconditional stability (9).

### 2.3 Model Boundary Condition Design

A multi-domain modeling approach (10) was used to characterize the hemodynamics, whereby the full 3D non-linear incompressible three-dimensional Navier–Stokes equations are used in the image-based CAD portion of the model, and a series of customizable 0D lumped parameter networks (LPN) are used to model the distal pulmonary and systemic circulations, as well as the patient’s collateral circulations and ventricular function, in a closed-loop manner. This 3D–0D multi-domain, closed-loop modeling approach has been previously used successfully to represent complex hemodynamics involving dynamic autoregulation (11, 12) and single-ventricle physiology (13). The CRIMSON Netlist Editor Boundary Condition Toolbox (NEBCT) enables the definition of complex cardiovascular LPN circuits and to easily account for atrial and ventricular septal defects, valve abnormalities, as well as abnormal collateral branches. All simulations were run under the assumption of rigid walls.

### 2.4 Model Boundary Condition Design and Parameterization

**Figure 2:**
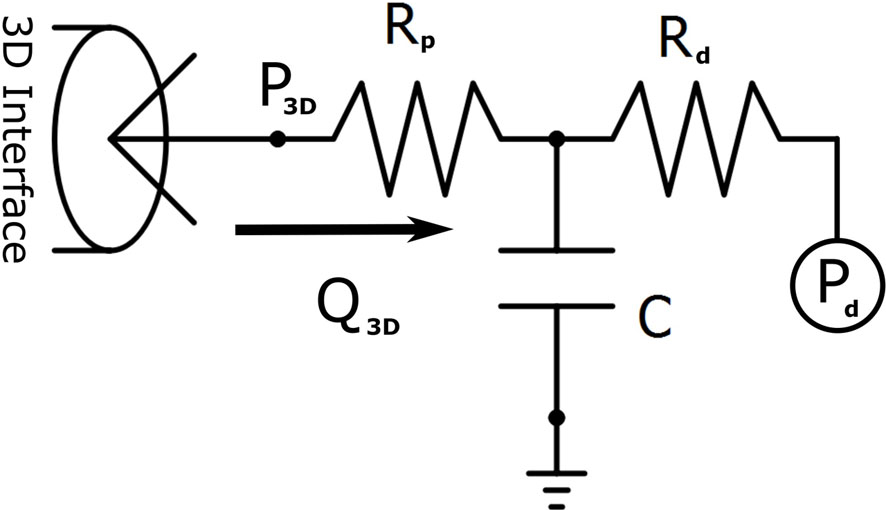
**The three-element Windkessel model, which is used to create boundary conditions at the vascular outflows of the three-dimensional domain**. Note that *P_d_* is given a fixed value in initial simulations, but once the full closed-loop circulation is created, it becomes a solution variable determined by the downstream venous system model to which it is connected.

Design and parameterization of the 0D LPN in order to achieve the full multi-domain closed-loop (10) model shown in Figure 1 proceeded in several stages. First, three-element Windkessel boundary conditions were applied at all outlet faces of the model (14), and the inflow at the neo-aortic valve was imposed according to PC-MRI flow recordings from the patient. The Windkessel model is shown in Figure 2; the equation for this model, relating pressure, *P*_3_*_D_*, and flow, *Q*_3_*_D_* at the three-dimensional interface, and with *C* the compliance of the vascular bed, *R_P_* the proximal resistance, *R_d_* the distal resistance, and *P_d_* the distal pressure, is given by

(1) $$\frac{{\mathrm{dP}}_{3D}}{\mathrm{dt}}{\mathrm{CR}}_{d}+P_{3D}-P_{d}+Q_{3D}(R_{d}-R_{p})-\frac{{\mathrm{dQ}}_{3D}}{\mathrm{dt}}R_{p}R_{d}C=0,$$

with suitable physiological initial values for *Q*_3_*_D_* and *P*_3_*_D_*, and with *P*_3_*_D_* fixed to zero in the initial simulations. Note that in the final complete closed loop, *P_d_* shown in Figure 2 becomes a solution variable determined by the state of the downstream vasculature model, as opposed to the fixed value that it was given during initial parameterization.

Iterative simulations were performed using the CRIMSON stabilized incompressible Navier–Stokes flow solver on 80 cores of the University of Michigan’s Flux High Performance Computing (HPC) cluster ConFlux, or 256 cores of a SGI UV 1000 HPC system at King’s College London. The resistances of the three-element Windkessel models were adjusted until the correct mean blood flow was observed at each outlet (targeting within 10% of the PC-MRI data), and the mean aortic root pressure matched the patient data. The Windkessel compliances were further tuned to approximately achieve the correct pulse pressure in the aortic root (targeting within 10% of the cardiac catheterization data).

**Figure 3:**
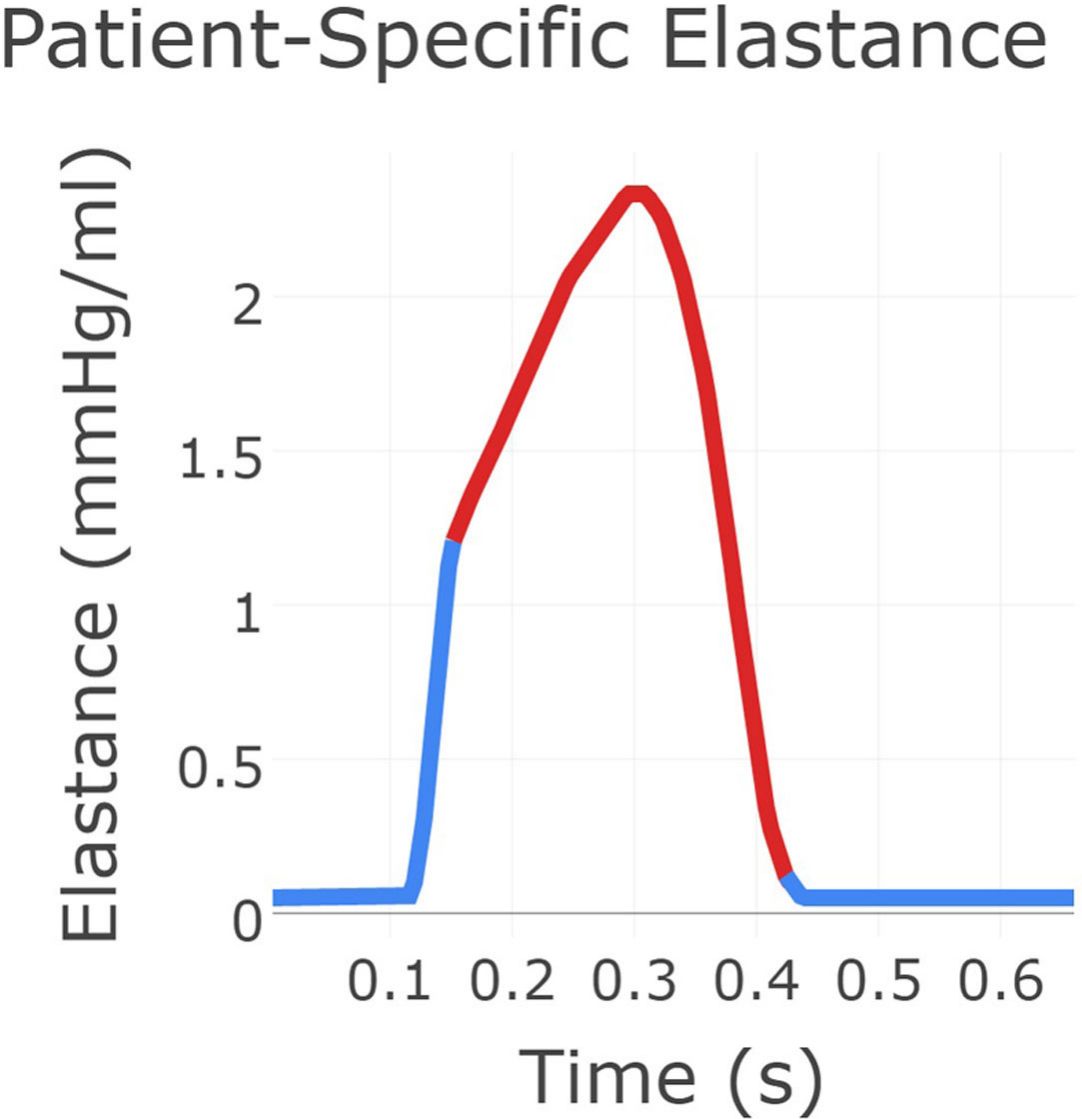
**The patient-specific time-varying elastance function created using a combination of data and exponential decay fitting**. The red region was derived from patient recordings of flow through the neo-aortic valve and continuous pressure recording in the ventricle. The blue region for which no data were available was produced using Gaussian extrapolation. This gives the time-varying relationship between pressure and flow in the patient’s ventricle.

Next, the flow through the neo-aortic valve was extracted from PC-MRI data, integrated in time and subtracted from the patient’s end-diastolic volume as given in the clinical report. This produced a systolic ventricular volume curve. This curve was aligned with the continuous pressure recording data taken inside the ventricle, and the pressure curve was point-wise divided by the volume curve. The result produced the patient-specific time-varying ventricular elastance during the period when the aortic valve is open. In order to complete the elastance function for the entire cycle, a single Gaussian curve was used to extrapolate the patient-specific systolic elastance on both sides. A Fourier smoothing was applied, ensuring that the interpolated function was *C*^1^-continuous throughout. Finally, the curve was scaled to ensure the peak elastance was not changed by the smoothing step. The resulting time-varying elastance function is shown in Figure 3. The red segment of the curve shows the patient-specific systolic portion of the elastance function, the blue corresponds to the Gaussian extrapolation of the remaining diastolic portion. A single-ventricle model with an atrial septal defect, informed by the clinical reports, was drawn using the CRIMSON Netlist Editor Boundary Condition Toolbox (NEBCT) (5–7) (see Figure 1), and the constructed elastance function was imposed on the ventricle using the CRIMSON Dynamic LPN Framework. Further LPN circuitry was drawn in the NEBCT, representing the inferior and superior vena cava, left and right pulmonary veins, and the aorto-pulmonary collateralization (Collaterals 1 and 2, Figure 1). The drag-and-drop circuit design tools of CRIMSON NEBCT permitted the creation of a physiological circulation, which would have otherwise been a difficult and arduous task. NEBCT also made straightforward any iterative redesign required during model development. The resulting closed-loop, single-ventricle circulation is shown in Figure 1.

The heart model was divided into a series of 0D components representing the right atrial, left atrial, and right ventricular compartments, including only the valves connecting directly to the single functioning ventricle. The atrial septal defect was modeled so that blood from the systemic venous and pulmonary venous circulation drains into the right ventricle through the tricuspid valve. The pulsatile contraction and relaxation of the right ventricle was modeled using the patient-specific elastance function described above, giving the time-varying ratio of the ventricular pressure to the difference between the ventricular volume and the unstressed ventricular volume (11). The valves were modeled via diodes that permit flow only in the forward direction. This heart model was connected to the neo-aortic valve surface of the ascending aorta. Due to the closed-loop approach, regional flows in the different systemic and pulmonary beds drain into a model of the venous system, thus enforcing the proper continuity of flow within the circulatory system. The final step of the parameterization was to fine-tune the closed-loop LPN component parameters to recover the hemodynamics (e.g., flow splits and pressures) observed in the individual. This required multiple iterative simulations, which were performed manually. While further tuning of the model parameters could have been performed, we were willing to tolerate some percentage of error, especially in cases where the absolute errors were small, since local small absolute errors have minimal global impact. The numerical values for parameters of all the LPN given in Figure 1 are summarized in Tables 1–3.

**Table 1:** **Parameter values for the three-element Windkessel models attached directly to the three-dimensional domain**.

	Rp	Rd	C
RC	0.50931	2.68885	0.71603
LC	0.50004	2.43319	0.74383
RS	0.50878	2.6307	0.74512
LS	0.45264	2.10615	0.84197
DAo	5.919 × 10^−2^	0.28437	5.01933
RPA	3.277 × 10^−2^	0.1361	1.76235
LPA	3.108 × 10^−2^	0.31950	1.00099

*Rp, proximal (closer to the aorta) resistance (Pa s mm^−3^); Rd, distal resistance (Pa s mm^−3^); C, compliance (mm^3^ Pa^−1^)*.

*See Figure 1 and its caption for the names and abbreviations of the vascular regions*.

**Table 2:** **Parameter values for the three-element Windkessel-like sections of the loop-closing circuit**.

	Rp	Rd	C
SVC	0.15500	1.807 × 10^−2^	284.73779
IVC	0.22075	2.261 × 10^−2^	135.95630
RPV	2.693 × 10^−2^	2.040 × 10^−2^	72.05470
LPV	1.027 × 10^−2^	8.970 × 10^−3^	132.96730

*Rp, proximal (closer to the aorta) resistance (Pa s mm^−3^); Rd, distal resistance (Pa s mm^−3^); C, compliance (mm^3^ Pa^−1^)*.

*See Figure 1 and its caption for the names and abbreviations of the vascular regions*.

**Table 3:** **Parameter values for the remaining (i.e., not covered by Table 1 or Table 2) sections of the loop-closing circuit**.

Component	Value
RA valve open resistance	3.3 × 10^−3^
RA valve inertance	6.667 × 10^−5^
Neo-aortic valve (NAV) open resistance	1.0 × 10^−3^
Neo-aortic valve (NAV) inertance	1.0 × 10^−5^
Collateral 1 resistance	9.69600 × 10^−2^
Collateral 2 resistance	0.05
Atrial septal defect resistance	3.0 × 10^−4^

*Resistances have units Pa s mm^−3^, inductances have units Pa s^2^ mm^−3^. See Figure 1 and its caption for the names and abbreviations of the vascular regions*.

### 2.5 Computational Derivation of the Percentage of Shunt Occlusion

During parameterization of the model, it was determined that it was impossible to achieve the patient data pressures recorded in the aorta and pulmonary arteries, while simultaneously achieving the patient-recorded flow through the BT shunt. This gave a fundamental indication that the resistance to flow of the BT shunt was no longer consistent with a BT shunt tube having the originally specified 3.5-mm diameter corresponding to the postoperative conditions. We determined that a suitable resistance could be achieved by reducing the shunt diameter by 22%; this corresponds to a 40% area occlusion in the shunt after 4 months. BT shunt stenosis or occlusion is a well-documented complication (15–17). Due to limitations in the resolution of the available MRI data, it was not possible to directly observe this degree of occlusion in the original dimensions of the shunt. Thus, this illustrates how computational simulations can enhance the available data on the individual without the need for further invasive assessments.

### 2.6 Examining the Hemodynamic Impact of an Alternative BT Shunt Design

**Figure 4:**
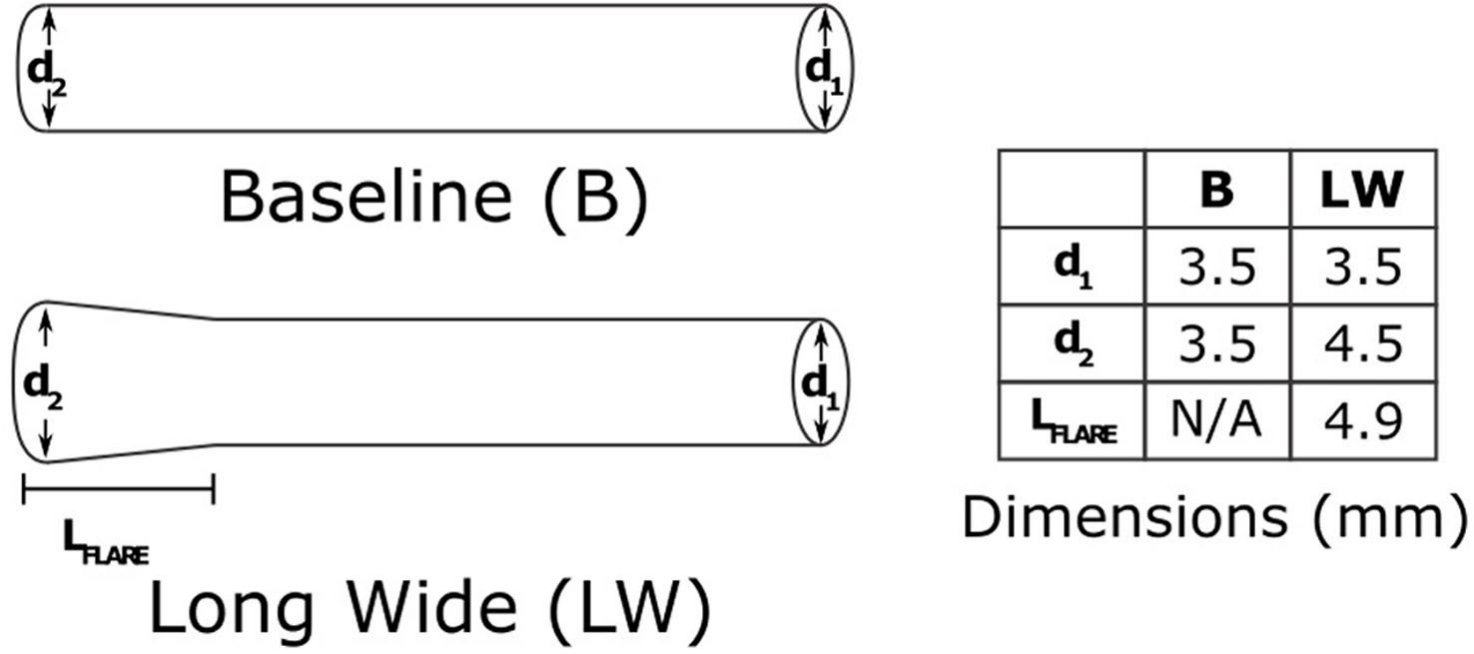
**The two shunt designs which we will consider in this paper**. The baseline shunt is the design that was used in the patient. The Long Wide flared shunt is a hypothetical alternative, which we evaluate numerically. The diameters are as they would be at the time of manufacture, without any occlusion. The shunts are both 14 mm in length.

Upon completion of a baseline model reproducing the patient’s clinical data, we used the model to investigate the impact of an alternative BT shunt on the hemodynamics in the pulmonary arteries. To achieve that, we adjusted the three-dimensional geometric model by replacing the original (cylindrical) shunt with a flared design, having nominal dimensions as shown in Figure 4. In light of the reduction in baseline shunt diameter described above, for the flared shunt, we also reduced all nominal diameters by 22%. After anisotropic mesh refinement, a finite element mesh of the geometric model of the Long Wide alternative shunt was created, consisting of 860,884 nodes and 4,707,343 elements. We then simulated the use of this shunt, without changing the parameters of the LPN circuit from those determined for the baseline case.

The impact of the different shunt designs on the hemodynamics in the PAs was evaluated by examining the resulting differences in mean pressure and mean flow in the left and right pulmonary arteries.

## 3 Results

### 3.1 Flow and Pressure Indices in the Baseline Shunt

**Figure 5:**
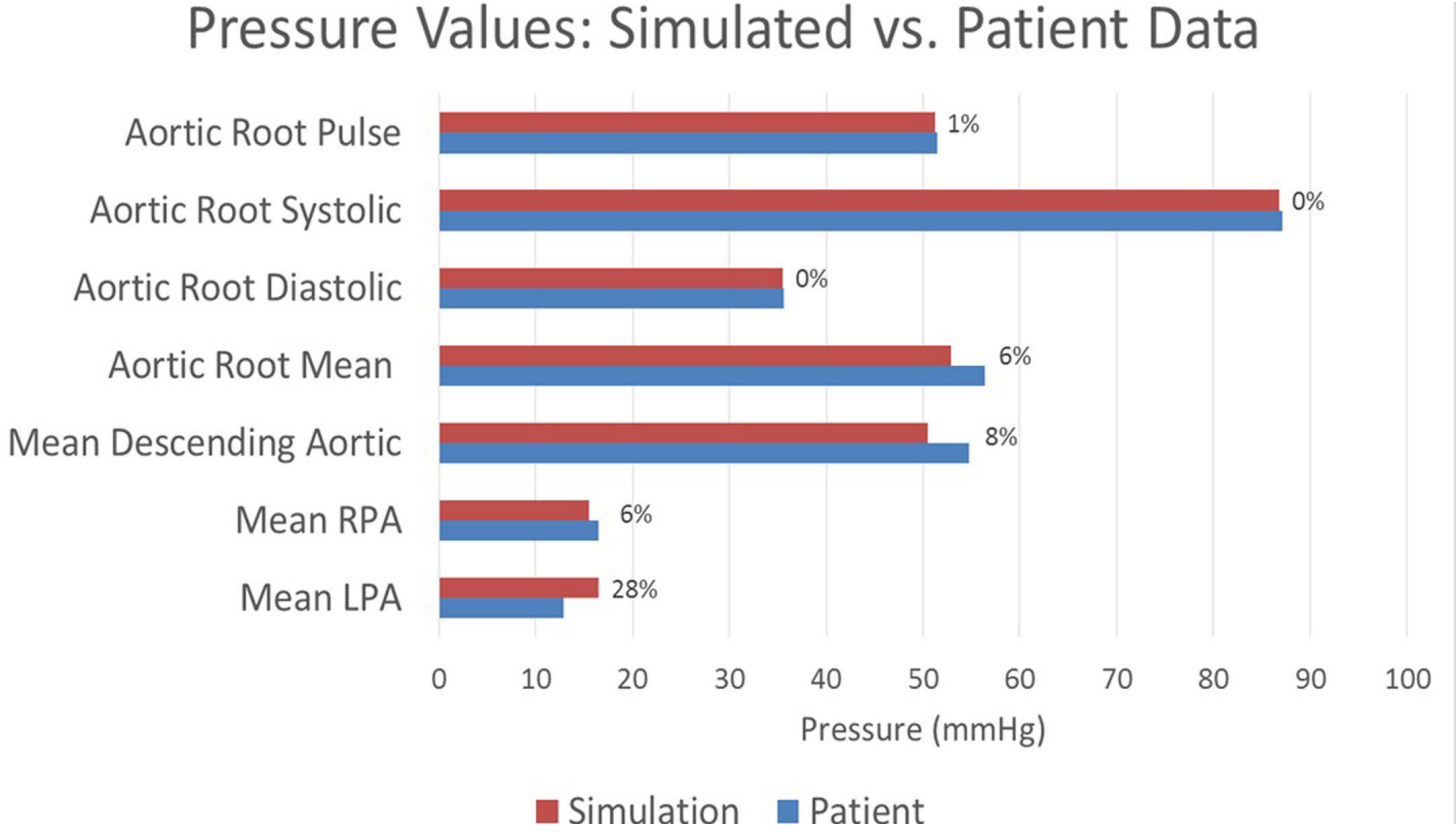
**A comparison between the pressures achieved in the baseline simulation and the pressures recorded in the patient at various locations throughout the model**. Each bar is marked with the percentage error in the simulated value relative to the patient recording. All assessed locations are visible in Figure 1.

**Figure 6:**
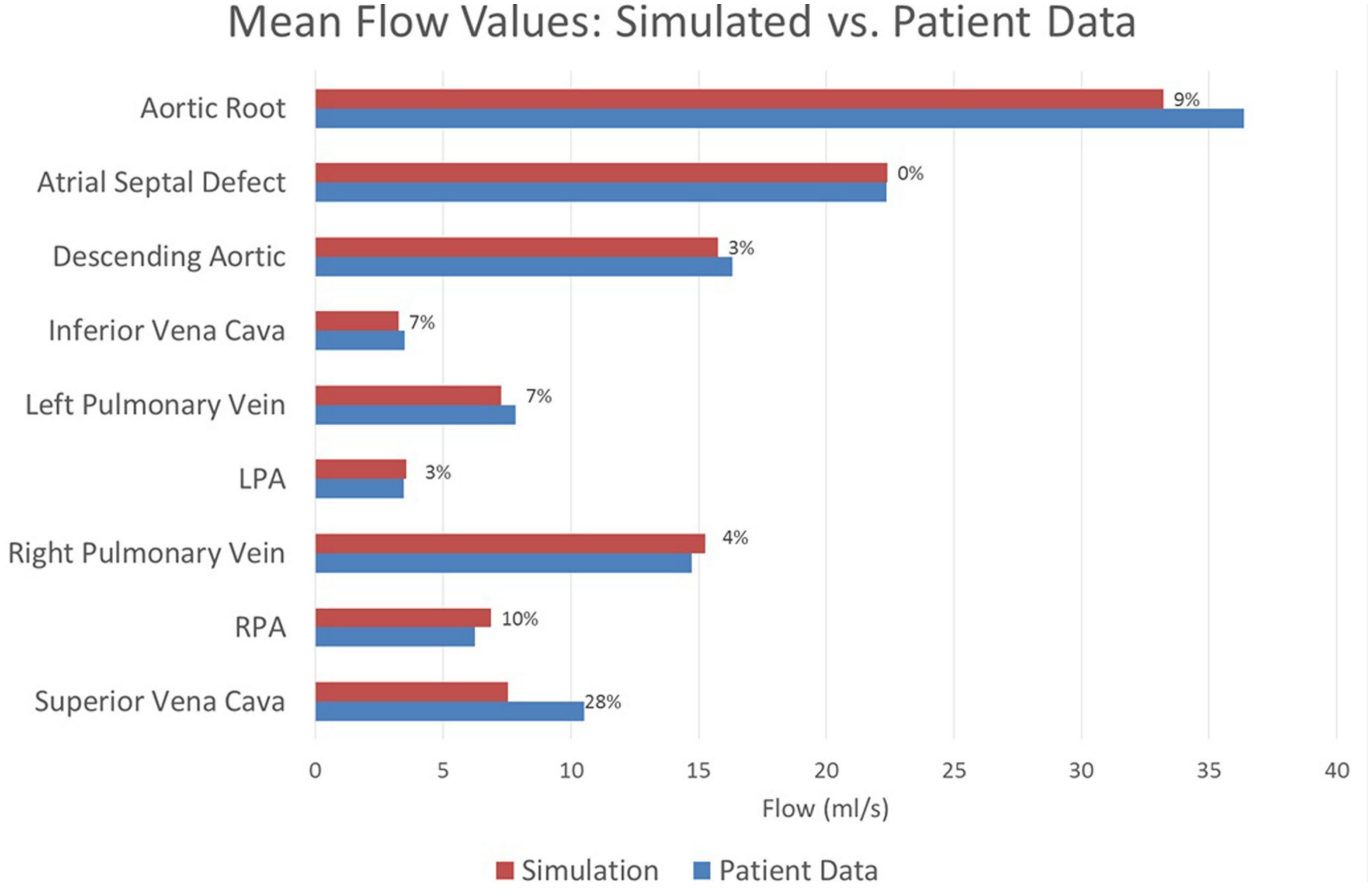
**A comparison between the flows achieved in the baseline simulation and the heart rate-adjusted flows from the patient at various locations throughout the model**. Each bar is marked with the percentage error in the simulated value relative to the patient recording. All assessed locations are visible in Figure 1. Note that the patient data for the atrial septal defect flow were computed by subtracting the patient data vena cava flow from the patient data cardiac output; all remaining patient data values were direct measurements.

The baseline shunt model successfully reproduced the patient’s hemodynamic data, given by the clinical report, the PC-MRI, and the cardiac catheterization data. Figures 5 and 6 show a comparison between computed and measured flow and pressure indices at different locations of the aorto–pulmonary circulation. Aortic root mean, pulse, systolic and diastolic pressures, and mean flow are recovered through the carefully adjusted patient-specific elastance function given in Figure 3. Fourteen of the 16 reported indices lie within 10% of the data. The biggest discrepancies are observed in the mean LPA pressure (28% error) and in the superior vena cava flow (27.8% error, which corresponds to a small absolute discrepancy of 2.92 ml/s). We note that despite the relatively large error in mean LPA pressure, both LPA and RPA pressures lie between the LPA and RPA mean pressure values recorded in the patient. Therefore, we claim that our model performs well in reproducing the hemodynamics for the baseline BT shunt case (with the noted 22% diameter reduction), indicating that we have created an accurate model and a solid foundation on which to perform further investigations into the impact of creating a flare on the distal end of the BT shunt. We remark that our baseline simulations indicate that the flow through Collateral 1 was 4 ml/s and the flow through Collateral 2 was 8.6 ml/s; comparable information was not available among the clinical recordings.

### 3.2 Comparative Impact of Shunt Flaring on Pulmonary Artery Hemodynamics

**Figure 7:**
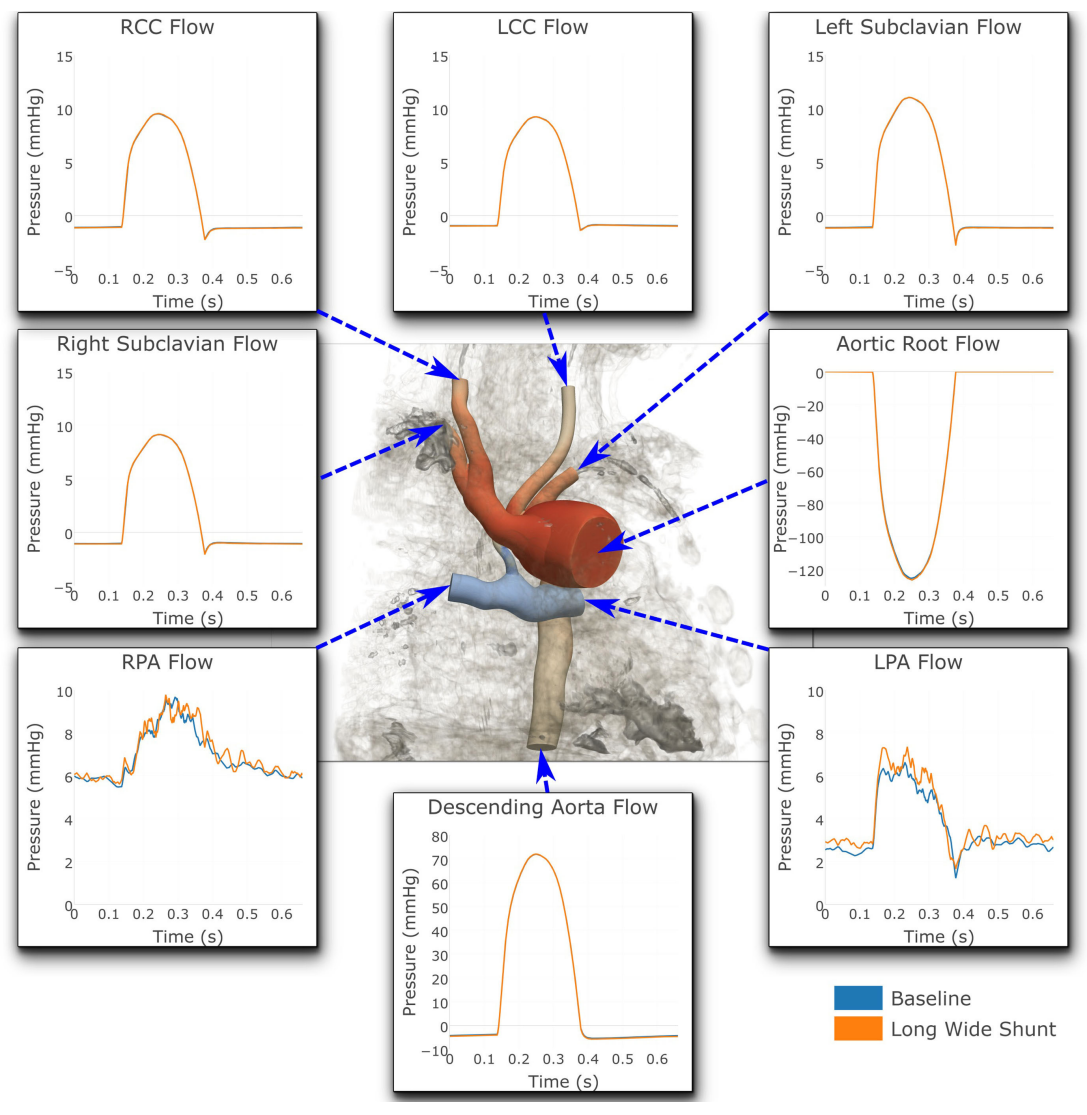
**Flows at all the boundaries of the three-dimensional domain**. At most locations, the flows are indistinguishable from one another. Note that three different *y*-axis scales have been used: one for the LPA and RPA, another for the aortic root and descending aorta, and a third for all the remaining outlets. For illustrative purposes, the geometric model has been embedded in a volume-rendering of the MRA image data, and a non-dimensionalized pressure coloring has been used on the surface of the geometry.

**Figure 8:**
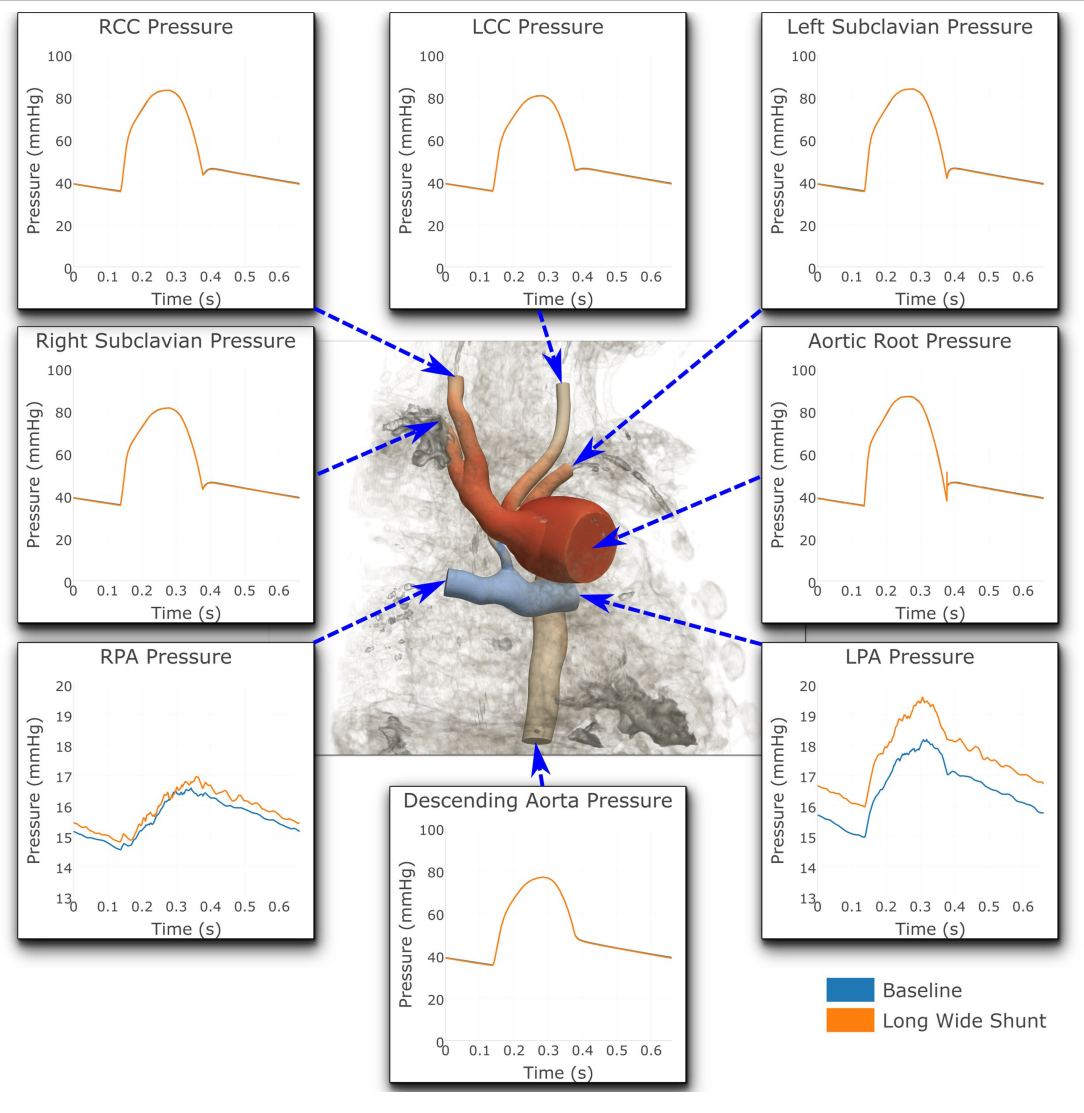
**Pressures at all the boundaries of the three-dimensional domain**. At most locations, the pressures are indistinguishable from one another. Note that two different *y*-axis scales have been used: One for the LPA and RPA, and another for all the remaining outlets. For illustrative purposes, the geometric model has been embedded in a volume-rendering of the MRI image data, and a non-dimensionalized pressure coloring has been used on the surface of the geometry.

Figures 7 and 8 show a comparison of the hemodynamics between the two studied shunt geometries, given in terms of time-resolved flow and pressure waveforms at each of the inlets and outlets of the three-dimensional model. The geometry is shown embedded within a three-dimensional volume-rendering of the original MRA data, providing anatomical context. We observe that suitable pressure and flow patterns are reproduced at all locations. In general, the pressure and flow traces at each outlet were not strongly affected by the shunt geometry, with the exception being the left and right pulmonary arteries. In the pulmonary arteries, we see that the waveforms for the two cases display different patterns of high-frequency oscillation within the data. This is likely indicative of the disturbed flow patterns within the pulmonary arteries being highly sensitive to the model geometry, due to the large Reynolds number flow through the BT shunt (in the baseline shunt, peak systolic Re = 1,830; shunt diameter 2.71 mm, peak systolic volumetric flow = 14,800 mm^3^ s^−1^, viscosity = 0.004 Pa s, density = 0.00106 g mm^3^). The maximum difference in LPA and RPA mean flows between the two models is 9%, and occurs in the LPA.

Figure 8 shows that the baseline shunt model presents lower mean pressure values in the LPA and RPA, of 16.51 and 15.55 mmHg, respectively. Conversely, the Long Wide flared shunt presents larger mean pressure values in LPA and RPA, 17.59 and 15.85 mmHg, respectively. This illustrates the impact of the smaller resistance to flow offered by the longer and wider flare compared to the baseline model.

A full numerical comparison of the impact of flaring on pressure and flow in the LPA and RPA is given in Table 4. We observe that flaring leads to an increase in mean pressure and flow in both the left and right pulmonary arteries, but there is considerable asymmetry in the effect; the LPA receives three times as much additional flow as the RPA with the flare present.

**Table 4:** **Breakdown of the change in hemodynamic parameters in the LPA and RPA, caused by the shunt flaring**.

	LPA pressure	RPA pressure	LPA flow	RPA flow
Baseline	16.5	15.6	3.6	6.9
Long wide	17.6	15.9	3.9	7.1
Change (%)	7	2	9	3

*Pressures are given in millimeters of mercury and flows in milliliters per second*.

### 3.3 Comparison of Shunt Properties

In Table 5, we see the impact of shunt flaring on pressure in and flow to the pulmonary arteries. Total flow to the pulmonary arteries increases by 6%, and mean pulmonary pressure increases by 4%. These results indicate that flaring may be beneficial if additional flow to the pulmonary arteries is required.

**Table 5:** **The impact of shunt flaring on pressure in, and flow to, the pulmonary arteries**.

	Baseline shunt	Long wide flared shunt
Mean aortic pressure (mmHg)	52.9	52.9
Mean pulmonary pressure (mmHg)	16.0	16.7
Shunt pressure gradient (mmHg)	36.9	36.2
Mean shunt flow (ml s^−1^)	10.4	11.0
Shunt resistance (mmHg ml^−1^ s)	3.5	3.3

## 4 Discussion

### 4.1 Determining Shunt Occlusion from the Computations

In the initial simulations using the originally specified dimensions of the BT shunt, we were unable to find a set of parameters which allowed reproduction of the clinical data. The best-case results obtained had mean aortic root pressure of 58.5 mmHg, mean pulmonary pressure of 38.6 mmHg, giving an approximate pressure gradient across the BT shunt of 19.9 mmHg, and mean BT shunt flow of 9.6 ml/s. This compared to the patient data indicating that the mean aortic root pressure should be 56.4 mmHg, the mean pulmonary pressure 14.7 mmHg, and the approximate pressure gradient across the BT shunt 41.7 mmHg, with a mean BT shunt flow of 9.7 ml/s. Thus, considering only the BT shunt, the simulation produced a 52% error in the pressure gradient estimate, while the error in the BT shunt flow was only 1%. This indicated that the resistance, and thus the assumed geometry of the BT shunt was incorrect. Using the Hagen–Poiseuille equation, we estimated that the diameter of the shunt should be reduced by 22% to achieve the correct resistance. Subsequent numerical simulations proved this estimate to be accurate, allowing us to recover the mean pressure in and flow to the pulmonary arteries with an error tolerance of 10%, compared to the previous 52% error. Specifically, with this geometry we achieved mean aortic root pressure of 52.9 mmHg, mean pulmonary pressure of 16.0 mmHg, giving an approximate BT shunt pressure gradient of 36.9 mmHg and a mean BT shunt flow of 10.4 ml/s. These values are summarized in Table 6, and correspond to errors relative to the patient data of 11 and 7%, respectively.

**Table 6:** **The disagreement between the patient data and the hemodynamic parameters of the baseline shunt when using implant-time dimensions and the agreement that was obtained by reducing the shunt diameter by 22% in our simulations**.

	Patient data	Shunt conforming to implant-time dimensions	Shunt with 22% diameter occlusion
Mean aortic pressure (mmHg)	56.4	58.5	52.9
Mean pulmonary pressure (mmHg)	14.7	38.6	16.0
Shunt pressure gradient (mmHg)	41.7	19.9	36.9
Mean shunt flow (ml s^−1^)	9.7	9.6	10.4
Shunt resistance (mmHg ml^−1^ s)	4.3	2.1	3.5

We have not directly confirmed that the diameter of the shunt has uniformly reduced by 22% along its length. Rather, we state that this or an equivalent change must have taken place in the patient in order to account for the observed results. An alternative scenario would be a greater level of narrowing at one end of the shunt, resulting in the same through shunt-equivalent resistance. Due to the resolution of the MRI images, we were not able to assess how the BT shunt geometry has truly changed in the patient.

### 4.2 Determination of Model Parameters

Significant effort was required during manual parameter tuning of our closed-loop LPN model. While the present work demonstrates the feasibility of such an approach, an important future development will be to begin using data assimilation techniques to determine these parameters algorithmically, and to a large extent, automatically (18, 19). This will be an important development, allowing us to examine and compare multiple such clinical cases.

### 4.3 Impact of Shunt Flaring on Pulmonary Artery Hemodynamics

The results show that there is potential for achieving increased pulmonary pressure and blood flow by using flared BT shunts, including changes in flow of up to 9% in the LPA. While it is clear that more pressure or flow is not automatically beneficial, such increases may have clinical value; given that a common complication after BT shunt placement is shunt stenosis, a shunt capable of delivering more flow may reduce the impact of the narrowing on PA hemodynamics (15–17). We believe that investigation of further alternative shunt designs is warranted. The flare we have used is relatively short, but we have demonstrated the feasibility and power of this modeling technique, laying a solid foundation for further investigation of alternative flared designs.

There may be limitations in terms of the widest possible flare, given the size of neonatal pulmonary arteries, but given that the largest diameter considered was 4.5 mm, and that the healthy 40-week gestation diameters for the main, left, and right pulmonary arteries has been reported to be 9.23, 5.65, and 5.49 mm, respectively (20), it is not inconceivable that the use of such flares may be clinically possible.

### 4.4 Pulmonary Artery Mean Pressure Discrepancy with Data

The clinical report indicated a pressure difference between the left and right pulmonary arteries of 3.6 mmHg. The largest such difference we could achieve computationally was 1.7 mmHg, which represents a significant discrepancy with the data. Most likely this indicates that some unknown factor, probably geometric, is not included in our model, although it may also be an error in the clinical measurement. For this reason, during parameterization, we chose to accept pulmonary artery pressures anywhere within the 3.6 mmHg range reported in the data (i.e., any values in the range 12.9–16.5 mmHg). Thus, the errors in the mean LPA and RPA pressures shown in Figure 5 should be understood within this context. Indeed, if we instead considered any simulated LPA and RPA pressure lying in this range to be of low error, then together with the 10% error tolerance used for all other values, the accuracy of our baseline simulation is even greater: fifteen of the sixteen reported indices considered in Figures 5 and 6 achieve or exceed our accuracy target.

### 4.5 Disturbed Flow in the Pulmonary Arteries and Numerical Accuracy

Flow within the pulmonary arteries was observed to be highly disturbed. This is apparent from the high-frequency oscillations which are present in the LPA and RPA pressure and flow waveforms, shown in Figures 7 and 8. By these measures, the degree of flow disturbance does not appear to be strongly dependent on the shunt geometry in the cases examined.

**Figure 9:**
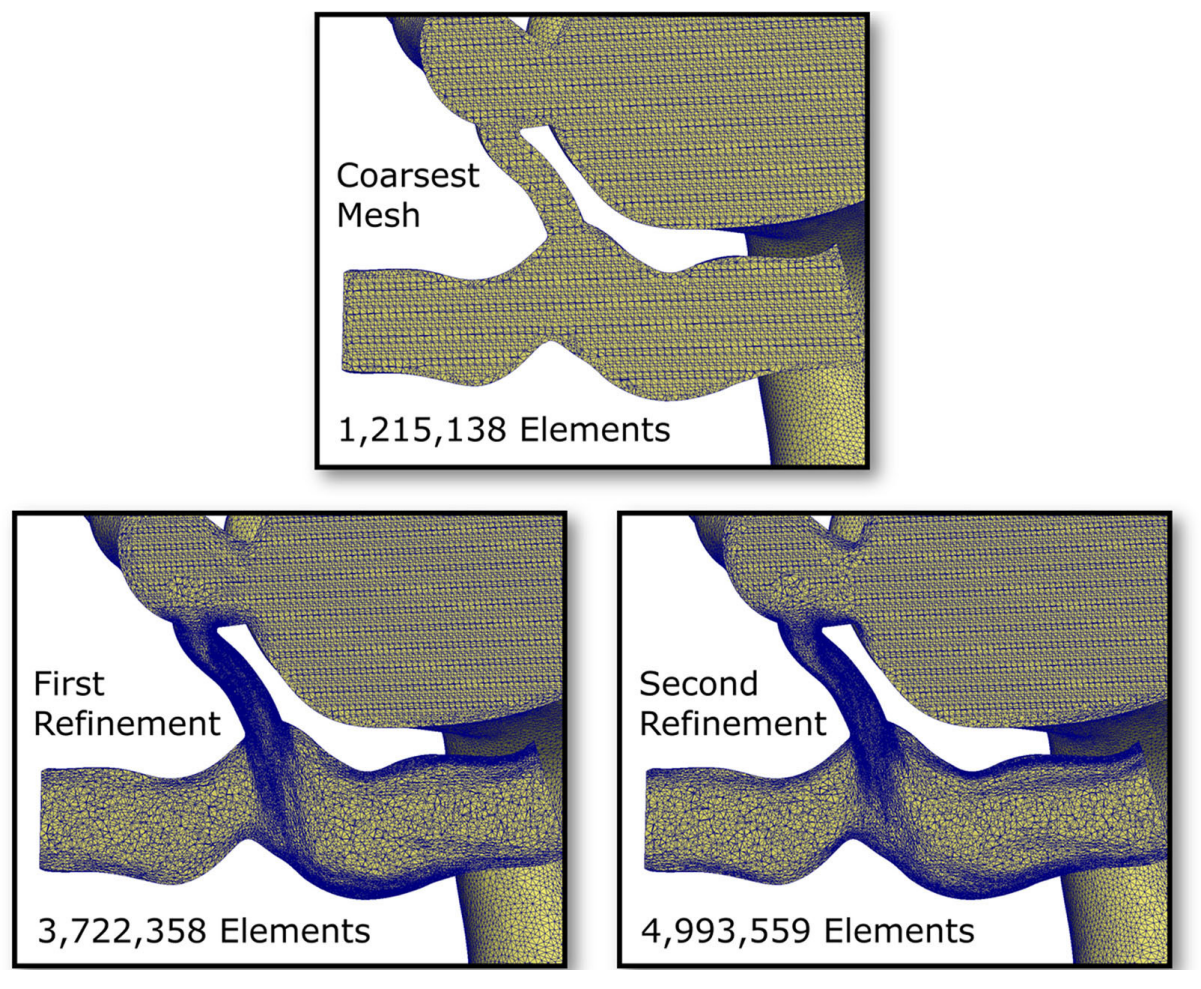
**Cut-through views of the finite element meshes within the pulmonary arteries and shunt in the baseline shunt design case**. Three levels of mesh refinement are shown; the refinements were generated algorithmically, based on a local velocity gradient error indicator (8). Notice the high level of refinement occurring in the shunt and the LPA.

**Figure 10:**
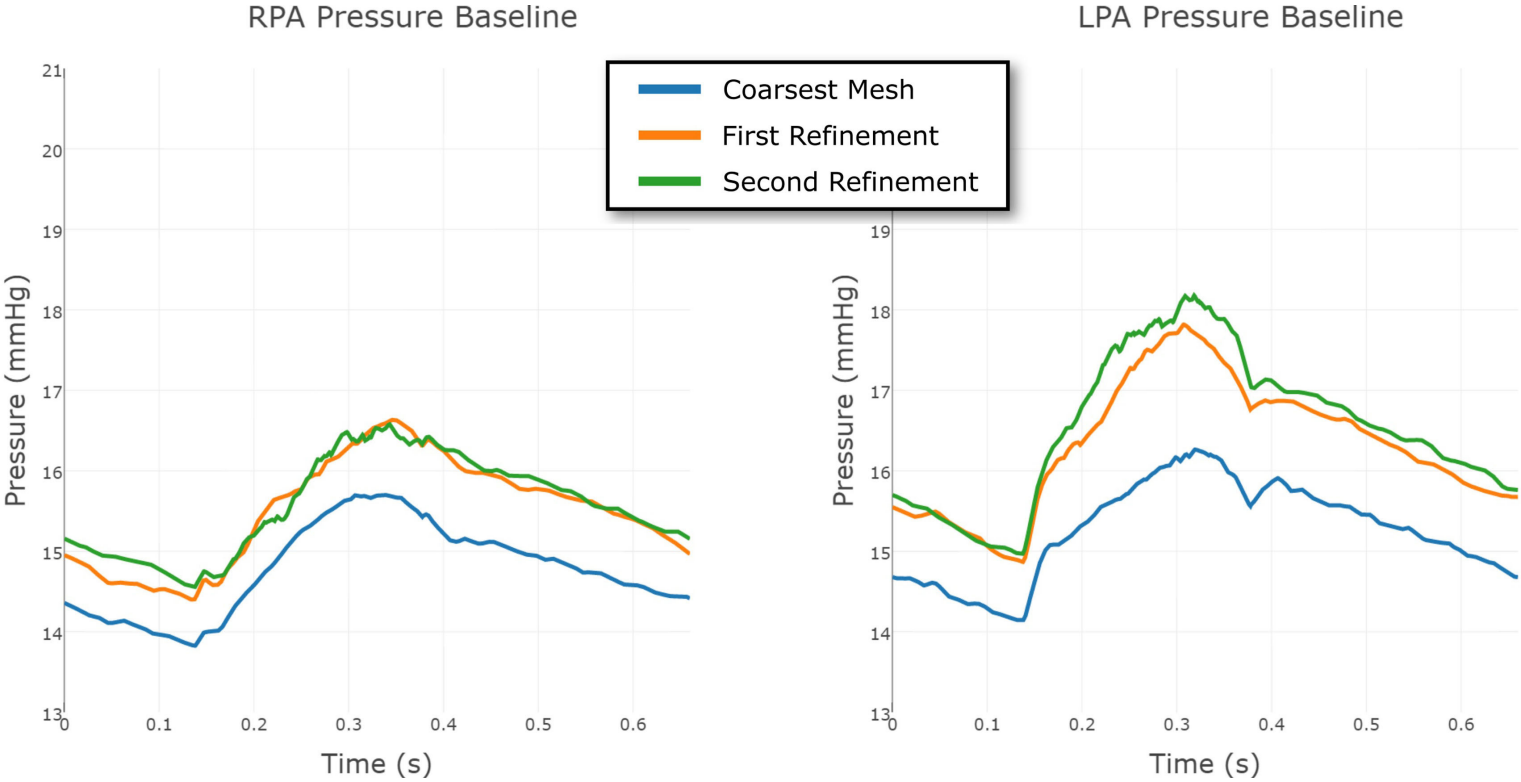
**Comparison between the pressure at the RPA and LPA outflows in the baseline shunt model, using three different levels of finite element mesh refinement**. The results are shown over a full cardiac cycle. The *coarsest mesh* consisted of 231,484 nodes and 1,215,138 elements; the *First Refinement* mesh consisted of 659,851 nodes and 3,722,358 elements; and the *Second Refinement* mesh consisted of 872,046 nodes and 4,993,559 elements. The mesh refinement is local, informed by an error indicator.

To eliminate the possibility of this oscillatory behavior being a numerical artifact, we attempted to eliminate numerical error by increasing the resolution of the finite element mesh. Mesh refinement was performed using an offline anisotropic adaptive meshing strategy, whereby a coarse mesh was generated, the simulation run, then a local error indicator based upon the hessian of the velocity field was used to perform mesh refinement (8). This strategy was repeated twice, resulting in three successively finer meshes, with the additional nodes and elements concentrated in regions of higher error. This can be seen in Figure 9, where we see that the shunt and LPA receives most of the refinement, whereas the mesh of the aorta and systemic vessels remains relatively unchanged. The pressure over one cycle in the LPA and RPA in the baseline shunt case with these levels of mesh refinement is shown in Figure 10. The *coarsest mesh* consisted of 231,484 nodes and 1,215,138 elements; the *First Refinement* mesh consisted of 659,851 nodes and 3,722,358 elements; and the *Second Refinement* mesh consisted of 872,046 nodes and 4,993,559 elements. The final results reported in this work were obtained using the *Second Refinement* mesh, and we see from Figure 10 that the level of convergence of the pressure waveform is sufficient (i.e., there is a reasonably small difference between results obtained with the second and first refinements), and that the oscillations do not reduce with mesh refinement.

**Figure 11:**
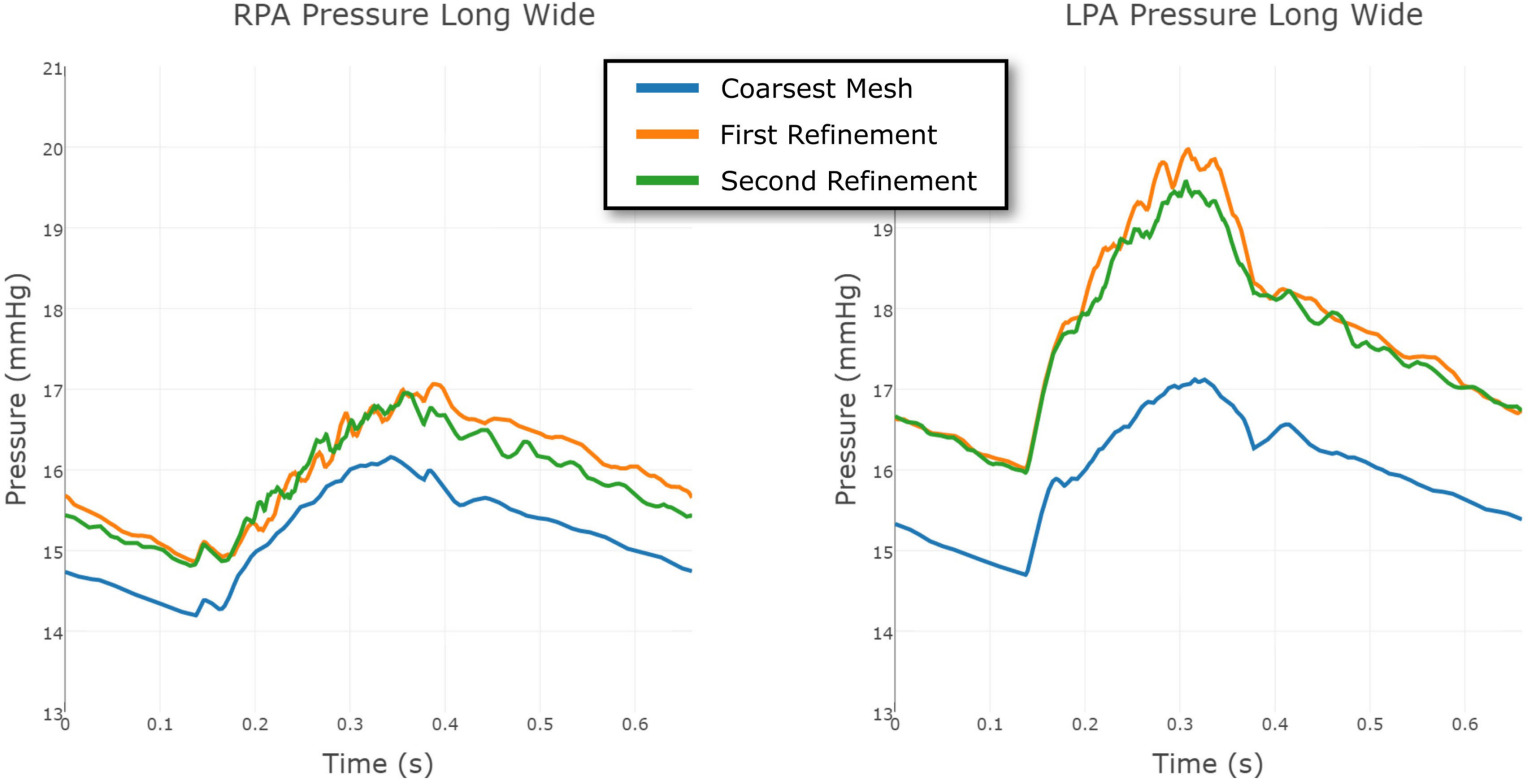
**Comparison between the pressure at the RPA and LPA outflows in the Long Wide shunt model, using three different levels of finite element mesh refinement**. The results are shown over a full cardiac cycle. The *coarsest mesh* consisted of 231,740 nodes and 1,216,259 elements; the *First Refinement* mesh consisted of 549,040 nodes and 2,919,044 elements; and the *Second Refinement* mesh consisted of 860,884 nodes and 4,707,343 elements. The mesh refinement is local, informed by an error indicator.

The same strategy was employed for the Long Wide shunt model. The three meshes, from coarsest to finest, had 231,740 nodes and 1,216,259 elements; 549,040 nodes and 2,919,044 elements; and 860,884 nodes and 4,707,343 elements, respectively. The convergence of the LPA and RPA pressure in this case is shown in Figure 11. The finest mesh was used for the results reported here, and this figure shows that the mesh is sufficiently refined for us to have confidence in the results.

**Figure 12:**
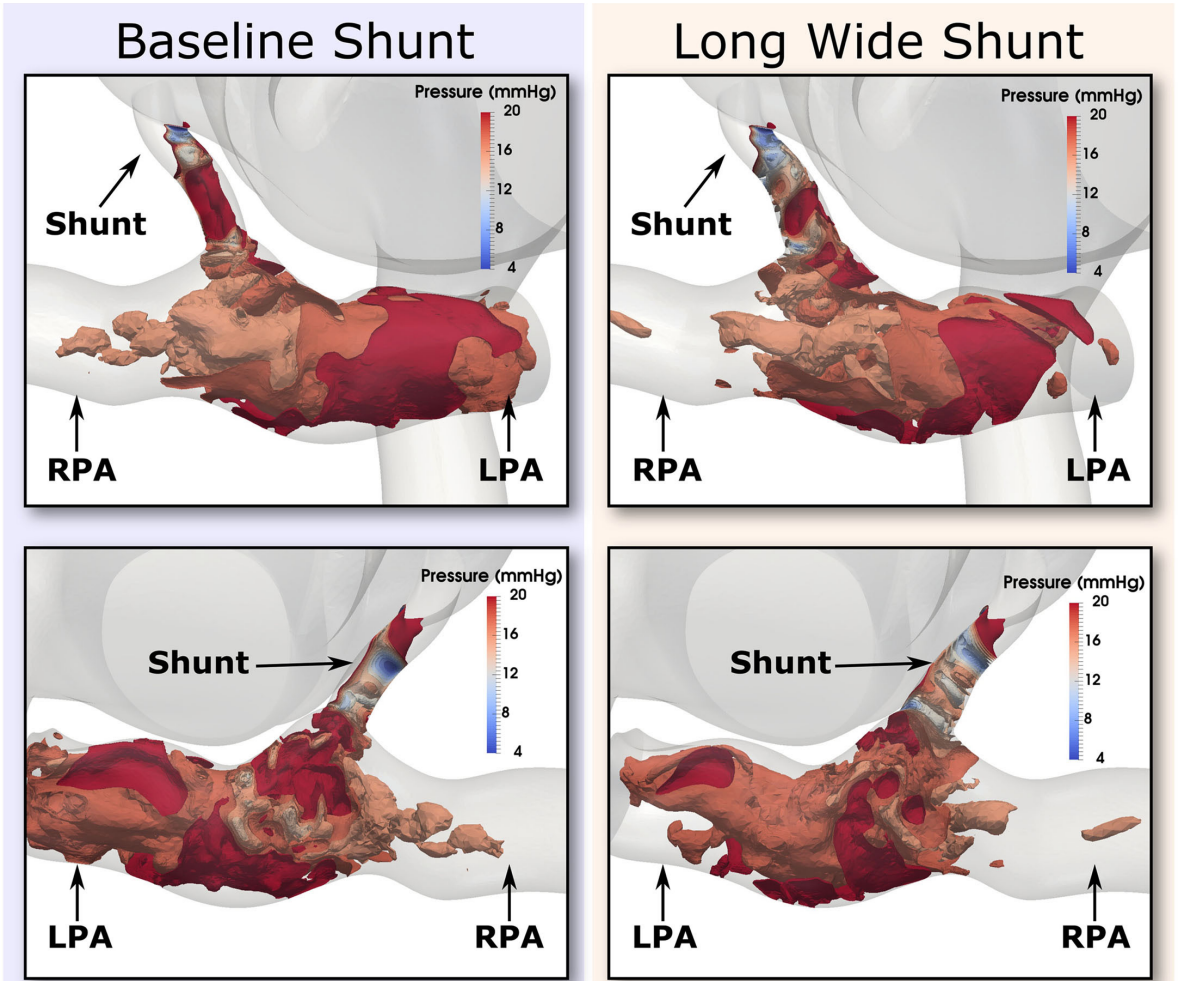
**Pressure isosurfaces at peak systole in the shunt and pulmonary arteries, with one isosurface every 2 mmHg in the inclusive range 4 and 20 mmHg**. Comparison is presented between baseline (left column) and the Long Wide alternative (right column) shunt. The views provided are approximately anterior (top row) and posterior (bottom row). Flow is highly complex in both cases.

Further evidence in support of the level of flow disturbance in the PAs is provided in Figure 12, where highly complex pressure isosurfaces at peak systole are shown for both the baseline and Long Wide shunt cases, using the most refined mesh for each. It is thus likely that the complexity of these flow patterns explains the high-frequency oscillations in the reported flow and pressure LPA and RPA waveforms. In a pulsatile flow such as this, it is possible that the maximum Reynolds number attained in the shunt (1,830) is associated with turbulence. Direct numerical simulations (DNS) would be required to further resolve the small scales of the flow and to properly quantify turbulence (21). Currently, there are no suitable mathematical turbulence models for pulsatile cardiovascular flows (22). However, this is beyond the scope of the present work.

To conclude, these results suggest that the oscillations are a genuine physical phenomenon, not uncharacteristic of the moderately high Reynolds numbers of the flow through the BT shunt (max. Re = 1,830). Further evidence for this being a physical phenomenon is given by the fact that the clinical pressure recordings taken in the PAs was observed to be qualitatively noisier than that taken in the aorta.

## 5 Conclusion

In the present work, we have demonstrated that we can reproduce the hemodynamics in a highly complex physiological case of a 4-month-old child who has a BT shunt, as measured by a rich patient dataset with sixteen points of comparison. The achieved fidelity to data was very good; as discussed above, fifteen of the sixteen indices examined can be considered to agree well with the patient data. The demonstration of the capability of this modeling approach to accurately and simultaneously reproduce many pressure and flow values is striking and will create a valuable starting point for further studies.

We were able to use simulations to enhance the information available in the MRI data and the pressure recordings. Specifically, we discovered that the resistance between the systemic circulation and the pulmonary arteries through the BT shunt was not what would be expected for a tube cleanly attached to each vessel and retaining its original effective internal diameter without any stenosis developing within it or near its points of anastomosis, and computing that the effective diameter of the BT shunt has reduced by 22% since the time of the initial surgery. This value corresponds to a 40% area reduction.

Examining the impact of flaring the shunt on the hemodynamics within the pulmonary arteries indicates that increases in PA pressures and flows are possible with the use of flared shunts. Further work should examine the impact of the shunt design on the wall shear stress and oscillatory shear index within the pulmonary arteries, as well as examining other hemodynamic indices of interest, such as platelet activation potential (PLAP), which may be relevant given the disturbed hemodynamics caused by the shunt (23–25). It should also be determined whether other alternate shunt designs can have further hemodynamic benefits. The work should be extended to investigate whether the same conclusions drawn here regarding the effective decrease in luminal diameter of the BT shunt also hold for other patients.

The creation of the highly complex simulation model (Figure 1), together with the modified versions with the different BT shunt designs, was enabled by the flexibility of our computational hemodynamics modeling and simulation package (5–7).

## Ethics Statement

This study has been performed with institutional review board (IRB) approval of the University of Michigan Health System. The title and ID of the protocol are “Assessment of Patient-Specific Hemodynamics Through Retrospective Clinical Data” (HUM00112350). Global IRB consent has been obtained to retrospectively analyze data for investigational studies using MRI images that have been anonymized.

## Author Contributions

CA wrote the paper, created the figures, developed the simulation tools, assisted in building the models, ran the simulations, and analyzed the results. PA built the geometric models, processed the data, wrote part of the paper, created figures, and ran simulations. AJ performed preliminary simulations and assisted in the preparation of the manuscript. AD and RG acquired the clinical data. RG and AF developed the concepts and assisted manuscript preparation. AF provided analysis of the simulation results.

## Conflict of Interest Statement

The authors declare that the research was conducted in the absence of any commercial or financial relationships that could be construed as a potential conflict of interest.
